# Molecular Dynamics Study of the Effect of Charge and Glycosyl on Superoxide Anion Distribution near Lipid Membrane

**DOI:** 10.3390/ijms241310926

**Published:** 2023-06-30

**Authors:** Xuan Meng, Huiyu Liu, Ning Zhao, Yajun Yang, Kai Zhao, Yujie Dai

**Affiliations:** 1 Tianjin Key Laboratory of Industrial Microbiology, College of Biotechnology, Tianjin University of Science & Technology, Tianjin 300457, China; mengxuantust@163.com (X.M.); liuhuiyutust@163.com (H.L.); 15620760988@163.com (N.Z.); 19974327683@163.com (Y.Y.); 2 Hebei Kingsci Pharmaceutical Technology Co., Ltd., Shijiazhuang 050035, China; 3 Jangxi Ourshi Pharmaceutical Co., Ltd., Xinyu 338012, China

**Keywords:** superoxide anion radical, membrane charge, molecular dynamics, glycolipid, lipid peroxidation

## Abstract

To examine the effects of membrane charge, the electrolyte species and glycosyl on the distribution of negatively charged radical of superoxide anion (·O_2_^−^) around the cell membrane, different phospholipid bilayer systems containing ·O_2_^−^ radicals, different electrolytes and phospholipid bilayers were constructed through Charmm-GUI and Amber16. These systems were equilibrated with molecular dynamics by using Gromacs 5.0.2 to analyze the statistical behaviors of ·O_2_^−^ near the lipid membrane under different conditions. It was found that in the presence of potassium rather than sodium, the negative charge of the phospholipid membrane is more likely to rarefy the superoxide anion distribution near the membrane surface. Further, the presence of glycosyl significantly reduced the density of ·O_2_^−^ near the phospholipid bilayer by 78.3% compared with that of the neutral lipid membrane, which may have a significant contribution to reducing the lipid peroxidation from decreasing the ·O_2_^−^ density near the membrane.

## 1. Introduction

Reactive oxygen species (ROS) [1,2,3] are some chemically reactive species containing oxygen, including hydroxyl radical (·OH), superoxide anion (·O_2_^−^), hydrogen peroxide (H_2_O_2_) and singlet oxygen (^1^O_2_), etc. ROS are produced in aerobic metabolism in vivo [4], which are derived from some endogenous sources such as mitochondria [5], peroxisomes [6], endoplasmic reticulum and phagocytic cells, etc. [7]. There exists an equilibrium between the production and elimination of ROS and the imbalance will result in oxidative stress. ROS has been implicated in the pathogenesis of certain diseases, including Alzheimer’s syndrome [8], atherosclerosis [9] and cancer [10]. In the case of Alzheimer’s disease (AD), ROS particularly affects neurons in the brain. This oxidative damage can promote the accumulation and aggregation of abnormal proteins, such as amyloid-beta plaques and neurofibrillary tangles, which are characteristic pathological features of AD [11]. Excessive ROS levels can oxidize low-density lipoprotein (LDL) cholesterol, converting it into oxidized LDL (oxLDL). OxLDL is taken up by immune cells, primarily macrophages, and leads to the formation of foam cells within the arterial wall [12]. Foam cells are a key component of early atherosclerotic plaques. Moreover, ROS can promote inflammation within the arterial wall, causing recruitment of immune cells and further plaque development [13]. In cancer development and progression, excessive ROS production can damage DNA, promoting genetic mutations that initiate the transformation of normal cells into cancer cells [14]. ROS-induced DNA damage can disrupt the normal control mechanisms of cell growth and division, allowing cells to evade normal growth constraints and acquire characteristics of cancer cells [15]. ROS can react with polyunsaturated fatty acids (PUFAs) to induce the release of toxic aldehyde metabolites such as malondialdehyde (MDA). MDA may be involved in cardiovascular diseases [16] and cancer [17]. This is due to the interacting of MDA with the functional groups of a variety of cellular compounds [18].

In addition to many experimental studies on lipid peroxidation between reactive oxygen species and phospholipids, the interaction between reactive oxygen species and phospholipid bilayer has also been studied theoretically. To examine the contact between ROS and phospholipid bilayer, Cordeiro [19] investigated the distribution, mobility and residence times of various reactive oxygen species at the membrane–water interface using molecular dynamics simulations with neutral POPC bilayer systems. It showed that molecular oxygen (O_2_) accumulated at the membrane interior. Conversely, superoxide (·O_2_^−^) radicals and hydrogen peroxide (H_2_O_2_) remained at the aqueous phase. In another research [20], he investigated the penetration and interaction of peroxynitrous acid with phospholipid bilayers. Yadav et al. [21] examined the neutral ROS such as H_2_O_2_, hydroxyl radicals (·OH), hydroperoxyl radical (HOO·), and O_2_ in native skin membrane (composed of ceramide, cholesterol, and free fatty acid in an almost equal molar ratio 1:1:1) using molecular dynamics. Additionally, the permeability of ROS was measured using free energy profiles (FEPs). The FEPs showed that in spite of high-energy barriers, ROS traveled through the membrane easily. However, these researches did not consider the charge interaction between and the charged radicals and phospholipid bilayer. Among ROS, ·O_2_^−^ is a key radical. In spite of its mild reactivity, it is ubiquitous in aerobic cells and plays an important role in the formation of other ROS such as ·OH, H_2_O_2_, ^1^O_2_ and ·ONOO^−^ (Someone calls the last as one in reactive nitrogen species (RNS)). RNS are various nitric oxide-derived compounds, including nitroxyl anion, nitrosonium cation, higher oxides of nitrogen, S-nitrosothiols, and dinitrosyl iron complexes [22]), which can induce oxidative damage in lipids, proteins and DNA [23,24]. The effects of ·O_2_^−^ can also be magnified when it produces other kinds of free radicals or oxidizing agents [25] (oxidizing agent is a substance that causes oxidation by accepting electrons and thus becoming reduced). Therefore, if the ·O_2_^−^ is inhibited, the damage caused by other free radicals will be then inhibited to some extent. Meanwhile, ROS accumulation will lead to lipid peroxidation and then cause cell damage. Chan et al. found that oxymyoglobin can improve the lipid peroxidation [26,27] and the extent of pro-oxidant effect is concentration-dependent [28]. Sotomatsu et al. demonstrated that synthetic dopa-melanin produced ·O_2_^−^ which could promote the peroxidative cleavage of phospholipids in the presence of Fe^3+^-ADP complexes [29].

In vivo, ·O_2_^−^ can be formed in neutrophil or some subcellular units, such as mitochondria [30] and peroxisomes [31]; all of the subcellular units mentioned above have lipid membranes. These membranes which are generally negatively charged can work in the environment surrounded by ·O_2_^−^. According to the hypothesis, the negatively charged membrane will repel the ·O_2_^−^ and reduce the contact between each other, which may reduce the ROS concentration near the phospholipid membrane, therefore it will affect the peroxidation of the phospholipid membrane. Although people think the negative surface charge of many cellular membranes concentrates protons and rarefies superoxide in their vicinity [32], it has not been evaluated in detail on the extent to which the membrane charge affects the superoxide anion distribution around.

With the improvement of computer performance and the development of molecular dynamics, there are many studies using molecular dynamics methods to study the ion distribution near the phospholipid layer. Takahashi et al. studied the distribution of counterions at the negatively charged lipid/water/air interface. It was shown that the thickness of the electronic double layer is different from the classical Debye length, which is a bit longer than the former. It was also found that the distance between the ions in the solution and the membrane charge changes depending on the difference in ionic charge owing to the water molecules around the ions [33]. Rodriguez and García studied NaCl ion distributions around DPPC lipid bilayers [34]. They find that Na ions directly coordinate with the DPPC lipid carbonyl groups. For low number of ions per lipid (1:16 and 1:8), most Na ions are bound to the lipid carbonyls, while the Cl form an ionic cloud around the lipid choline groups. However, there is no report on the research of the distribution of charged radical ·O_2_^−^ near the charged lipid bilayers.

Additionally, the lifespan of different cells in animals varies greatly, among which nerve cells survive much longer than other ordinary cells. Surely, many factors determine the lifespan of a cell. In non-dividing cells [35], essential components that become damaged cannot be diluted out through cell division but must, of necessity, be turned over and renewed. By elevating stress resistances, many of the activities needed for such renewal should be elevated with commensurate reduction in the steady state levels of damaged cell components. Therefore, chronological lifespan in particular might be expected to relate to stress resistance [36]. In terms of membrane structure, the significant difference between membranes of nerve (or brain) and other ordinary cells is the richer glucolipids, such as gangliosides GM1, GM2 and GM3 [37]. Some people have studied the protective effect of glycolipids on the membrane, among which have shown that adding foreign glycolipids can significantly reduce the lipid peroxidation of keratinocytes [38]. Of course, the protective effect of sugar is multifaceted, but there is no doubt that the presence of sugar on the surface of the membrane makes it difficult for ROS near the surface of the glycolipid membrane to effectively approach the phospholipid bilayer owing to the physical obstruction of sugar groups. Therefore, the effects of charge and sugar on the distribution of ROS near the phospholipid bilayer are one of the perspectives from which to study their protection effects on cell membrane. Considering that the activities of different ROS are greatly different, the concentration of high active hydroxyl radical is very low, and almost has no selective reaction with the surrounding organic compounds, it is difficult to simulate the equilibrium state of this kind of free radical. ·O_2_^−^ is relatively stable, it has a longer life with a long distance of movement and it has various ways to convert to active hydroxyl radical or peroxynitrite anion near the phospholipid bilayer to cause lipid peroxidation. Therefore, considering the distribution of ·O_2_^−^ near phospholipid bilayers and its influencing factors is of great significance for reducing the harm of ROS and controlling lipid peroxidation. However, previous studies have mainly focused on the interaction between neutral radicals and cell membranes, as well as their distribution in neutral membranes. In the case of biological cell membranes and organelle membranes, which are negatively charged, there must be an electrostatic interaction between negatively charged ROS such as ·O_2_^−^ and negatively charged cell membranes. However, so far, no one has considered the influence of electrostatic interactions between charged radicals and charged phospholipid membranes on the distribution of radicals near the phospholipid membrane. Hence, in order to clearly understand the impact of membrane charge, temperature, electrolytes in aqueous, and glycolipids on the distribution of ·O_2_^−^ near phospholipid membranes, neutral and negatively charged phospholipid membrane systems containing superoxide anions were constructed, respectively. Molecular dynamics simulations were performed at different temperatures of 310 K and 330 K to compare the effects of membrane charge and temperature on the distribution of ·O_2_^−^ near the membrane. Furthermore, NaCl and KCl electrolytes were added separately to examine the influence of different electrolytes on the distribution of ·O_2_^−^. Finally, glycolipids were introduced to construct a phospholipid bilayer system containing glycolipids, and the impact of surface sugar moieties on the distribution of ·O_2_^−^ was investigated.

## 2. Results and Discussion

### 2.1. The Pathway of Transformation of ·O_2_^−^ to Hydroxyl Radical

The superoxide anion free radical (·O_2_^−^) is a common reactive oxygen species in the body [39]. It differs from other reactive oxygen species in that it carries a negative charge. Due to its lower reactivity and longer lifespan compared to hydroxyl radicals, ·O_2_^−^ is widely distributed in the body. Although it has low reactivity in inducing lipid peroxidation by directly reacting with fatty acid chains, it can mediate lipid peroxidation by converting to hydroxyl radicals (·OH), which are the main reactive oxygen species involved in lipid peroxidation. The conversion of ·O_2_^−^ to hydroxyl radicals occurs through several mechanisms:

(1) Hydroxyl radicals are primarily produced via the Haber–Weiss reaction of excess superoxide anions and hydrogen peroxide [40]:

(1) $$\cdot \;\;O_{2}^{-}+H_{2}O_{2}=\cdot \;OH+OH^{-}+O_{2}\;$$

(2) Another pathway involves the reaction of superoxide anions with hypochlorous acid (HOCl), which is released by immune cells such as neutrophils. This reaction also generates hydroxyl radicals [41]:

(2) $$\cdot \;O_{2}^{-}+HOCl=\cdot \;OH+Cl^{-}+O_{2}\;$$

This mechanism is used by neutrophils for their attack against microbes and tumor cells [42,43]. Bauer described in detail the complex relationship of HOCl and ·O_2_^−^ related to cell attack and protection. He pointed out that the HOCl and ·O_2_^−^ concentration near the membrane surface are essential for the cell apoptosis [44].

(3) ·O_2_^−^ can also react with the endogenous NO to produce ONOO^−^ radical, ONOO^−^ can then protonate to form peroxynitrous acid, which homolyzes to yield nitrogen dioxide (NO_2_) and hydroxyl radicals (·OH) [45]:

(3) $$•\;O_{2}^{-}+NO=•\;ONOO^{-}$$

(4) $$•\;ONOO^{-}+H^{+}=•\;ONOOH\;\;$$

(5) $$•\;ONOOH=NO_{2}+•\;OH\;$$

### 2.2. The Main Form of Superoxide Anion Radical in Aqueous Solution

Hydroxyl radicals are highly reactive and rapidly react with organic compounds they encounter, but they can only travel short distances in vivo. Therefore, to participate in lipid peroxidation, hydroxyl radicals need to be produced in the vicinity of the membrane. In contrast, superoxide anions can travel longer distances to reach the membrane. Additionally, there is an ionization equilibrium between ·O_2_^−^ and its protonated form, ·HO_2_ [46].

(6) $$•\;O_{2}^{-}+H^{+}=•\;{\mathrm{HO}}_{2}$$

At physiological pH, this equilibrium has a pKa of 4.7 [47], which means that only 0.2% of all ·O_2_^−^ is in •HO_2_ form in the aqueous phase. The ionic state enhanced its water solubility. The concentration of •HO_2_ near the membrane surface depends on its equilibrated ·O_2_^−^ concentration, and a relatively high concentration of ·O_2_^−^ radicals near the membrane will obviously promote the •HO_2_ concentration.

### 2.3. The Dynamic Equilibrium of the Systems

From the analysis above, to study the distribution of ·O_2_^−^ near the membrane is of great significance for lipid peroxidation. Meanwhile, whether the membrane charge, temperature, species and concentration of electrolytes near the membrane have effects on the distribution of ·O_2_^−^ near the lipid membrane needs to be examined. Further, whether the sugars in glycolipids significantly affect superoxide anion contact with hydrocarbon groups is also an interesting question. Thus, the phospholipid bilayers, including the neutral lipid membrane composed POPE, the negatively charged membrane composed of POPE and POPG, and glycolipid bilayer having GM1 and GD1a were constructed through the Charmm-GUI webserver. The forcefield of ·O_2_^−^ was constructed by Amber16 and G09 software. The systems containing different electrolyte, ·O_2_^−^ radicals and phospholipid bilayers were built with Amber16. All systems built were equilibrated with molecular dynamics by using Gromacs 5.02. The tcl code written by us running in VMD package is used to calculate the statistical behavior of different particles near the lipid membrane. Because most superoxide anions exist in the equilibrium system in the form of deprotonated ions, only superoxide anions are considered in molecular dynamics in this study. In order to examine the density of ·O_2_^−^ near the membrane in an equilibrium state, the systems constructed need to be sufficiently dynamically balanced. The composition of eight phospholipid bilayer systems constructed with different dynamics temperature (T) for investigation was listed in Table 1.

**Figure 1:**
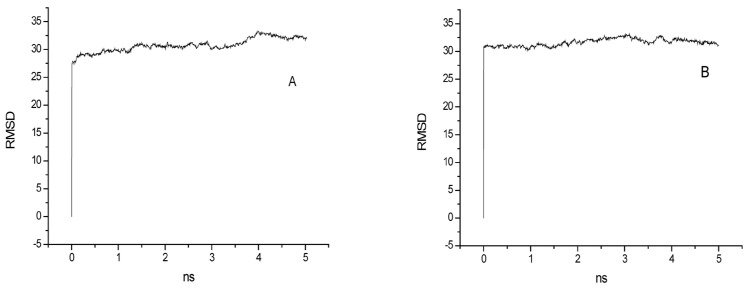
RMSD changes with the time during the MD simulations for systems (**A**,**B**).

To better characterize membrane structural changes within MD systems, VMD1.91 was used to analyze the RMSD changes with the dynamic.s simulation, which specify the instantaneous orientation of the molecules. The NPT dynamic equilibrium process of each system was performed at intervals of 5 ns until the RMSD change of the last interval tended to approximate a straight line. Subsequently, the production process continued to run and the last 5 ns dynamic trajectory file of each system was statistically analyzed. The RMSD changes of the lipid molecules of different systems (systems A and B) during the MD process are shown in Figure 1. The RMSD images of other systems were not shown.

### 2.4. Effects of Different Charges on ·O_2_^−^ Density near the Lipid Membrane Surface

**Figure 2:**
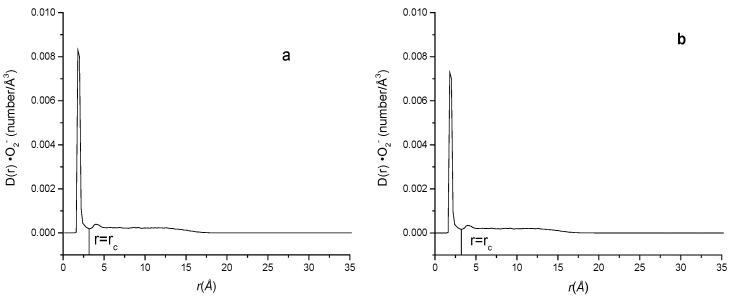
·O_2_^−^ distribution at different distances from the phospholipid membrane surface in the two systems ((**a**): system A; (**b**): system B).

There are a lot of papers study the ion distribution near the cell membrane [33,48,49,50]. It can be seen that the common ions such as Cl^−^ and Na^+^ are not evenly distributed near the lipid membrane. There exists a maximum density peak near the membrane surface, indicating that the enrichment phenomenon is present for many ions near the membrane. To examine the effect of membrane charge on the approaching of ·O_2_^−^ to phospholipid membrane, a neutral bilayer composed of neutral phospholipid POPE and a negatively charged phospholipid bilayer composed of POPE and the negatively charged phospholipid POPG with a molar ratio of 3:1 (ne:ng = 3:1) were constructed, respectively. The density curves of ·O_2_^−^s at different distances from the membrane surfaces of the neutral and negative charged membrane systems (Systems A and B) are shown in Figure 2. It can be seen that similar to the electrolyte ion distribution near the membrane reported by others [51], the superoxide anion density distribution also has a maximum peak near the membrane surface. The enrichment of superoxide anions near the membrane surface greatly increases the probability of hydroxyl radical generation and lipid peroxidation near the membrane. Although the concentration of ·O_2_^−^ in actual biological systems is much lower than in the simulated systems of this study, it is still important to consider that any factor reducing the contact between ·O_2_^−^s and the membrane would be beneficial in reducing lipid peroxidation.

To give an obvious comparison about the probability difference of ·O_2_^−^s appeared near the negatively charged and neutral membranes, the percentage ratio of the peak area within the first peak from the surface (r < 3.2 Å for ·O_2_^−^) of the membrane to the total area of the curve, $R_{•O_{2}^{-}}$, which represents the probability of ·O_2_^−^s appeared near the membrane surface was calculated as Equation (10) and listed in Table 1.

(7) $$R=\frac{A_{r}}{A}\%\;$$

where *r* is the distance of ions to the membrane surface, *A_r_* is the area of the first peak of the ions representing the probability density of ions near the membrane surface and A is the total area below the curve. The values of *A_r_* and *A* are obtained using Origin 7.5 from the integration of the probability density distribution curve as Figure 2 from r = 0 to r = r_c_ that the first minimum of D(r) appeared after the first peak. r_c_ for different components of ·O_2_^−^, K^+^, Na^+^ and Cl^−^ in the aqueous phase of systems A to H are 3.2 Å,3.2 Å,2.8 Å and 3.8 Å, respectively.

**Figure 3:**
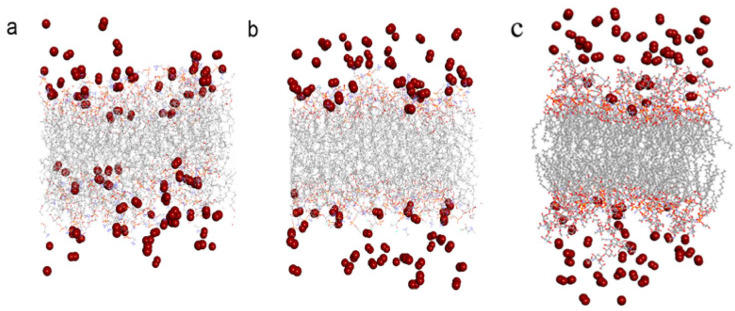
·O_2_^−^ distribution in the neutral and negatively charged phospholipid membrane systems A (**a**), B (**b**) and H (**c**).

It can be seen that the probability of ·O_2_^−^s within 3.2 Å of the membrane surface in neutral membrane system (System A), $R_{•O_{2}^{-}}(A$) and negatively charged membrane system (System B), $R_{•O_{2}^{-}}(B$) were different. That is, in the neutral charged membrane system, $R_{•O_{2}^{-}}(A$) was 56.89%, while in the negatively charge membrane system, $R_{•O_{2}^{-}}(B$) was only 35.26%. Compared with the neutral membrane system, $R_{•O_{2}^{-}}$ near the membrane surface was reduced by 38.02% ((56.89–35.26%)/56.89%) in the negatively charged phospholipid membrane, indicating that the occurrence probability of ·O_2_^−^ near the negatively charged phospholipid membrane is reduced by 38.02% compared with the neutral phospholipid membrane. Therefore, it may reduce the probability of other active free radicals derived of superoxide anions (such as ·OH) appearing in the vicinity of the membrane, thus reducing the attack of the active free radicals on the lipid components of the membrane. Figure 3 shows the appearance view of ·O_2_^−^ distributions in the systems containing neutral and negatively charged membranes (Systems A and B), respectively, at a certain time of the equilibrated state. It can be seen that there are more ·O_2_^−^s appearing close to the membrane surface in the neutral phospholipid membrane system than that in the negatively charged membrane system. The occurrence probability (R values) of the different ions such as ·O_2_^−^, Cl^−^, K^+^ and Na^+^ near the phospholipid membrane surface in different systems were listed in Table 2.

**Figure 4:**
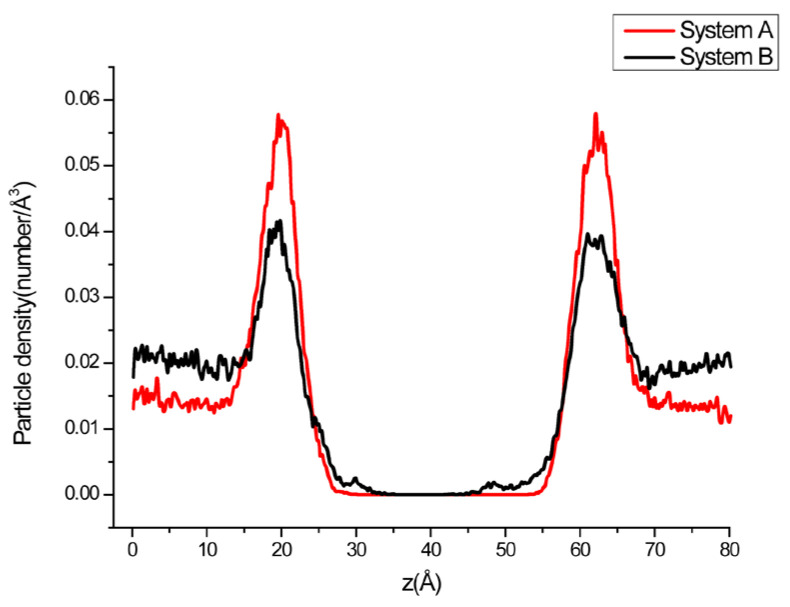
Density profiles of ·O_2_^−^ across the lipid bilayer for systems A and B.

Figure 4 shows the distribution of different components along the direction perpendicular to the membrane (z-axis) in system A and B. It can be clearly seen that the ·O_2_^−^ density peak of system B is significantly lower than that of system A, and the distribution of ·O_2_^−^s system B at the far end of the membrane is higher than that of A.

The reason for this phenomenon can be attributed to the negative charge on the sur-face of the bilayer containing POPG, which repels the negatively charged superoxide anions, changes the number ratio of anions near the membrane, and reduces the distribution of negatively charged ·O_2_^−^s near the negatively charged phospholipid membrane. Thus, the probability of generating hydroxyl radicals which can undergo lipid peroxidation near the phospholipid bimolecular membrane is reduced.

**Figure 5:**
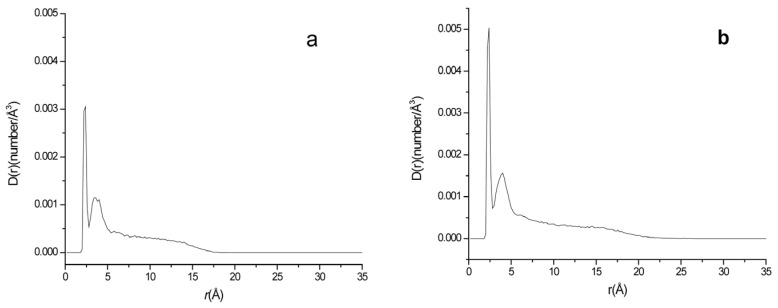
Density of K^+^ at different distances from the phospholipid membrane surface in the systems A (**a**) and B (**b**).

In addition, we listed the density curves of K^+^ at different distances from the surface of phospholipid membranes in both systems (A and B). As shown in Figure 5, the K^+^ curves show obvious bimodal distribution. In the phospholipid bilayer systems, the radial distribution of electrolyte ions exhibits a bimodal phenomenon, as observed in the literature. However, the specific reasons for this phenomenon are currently not well understood. Further research is needed to investigate and elucidate the underlying mechanisms responsible for the bimodal distribution of electrolyte ions in these phospholipid bilayer systems. The first peak appeared from 1.6–3.2 Å while the maximum value of K^+^ occurrence density appeared at 1.8 Å and 2.0 Å for systems A and B, respectively. However, there are higher K^+^ occurrences ($R_{K^{+}})$ within 3.2 Å from the membrane for system B (23.83%) than that for system A (21.97%) (Table 2) due to the attraction of the negatively charged phospholipid membrane. Considering that the system is electrically neutral, although the negatively charged phospholipid membrane (System B) repels some ·O_2_^−^s away from the membrane surface, the overall negative charge near the membrane (from POPG and a small amount of ·O_2_^−^s) is more than that near the neutral phospholipid membrane, so there are more balanced cations near the negatively charged phospholipid membrane than that near the neutral membrane.

### 2.5. Effects of Different Temperatures on ·O_2_^−^ Ions Density near the Lipid Membrane

**Figure 6:**
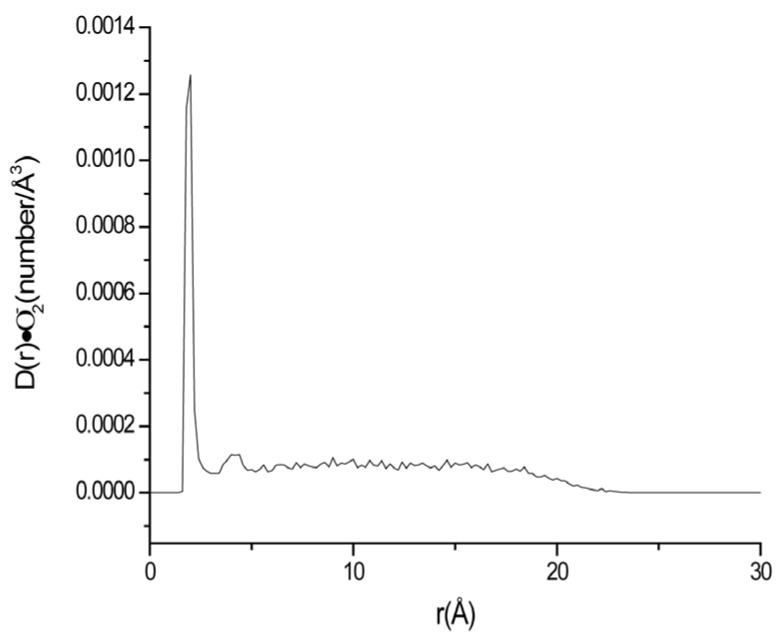
Density change of ·O_2_^−^ at different distances from the surface of negatively charged lipid membrane system C at 330 K.

In order to examine the effects of temperature on the distribution of superoxide anion near the membrane, the negatively charged membrane system at 310 K (System B) and 330 K (System C) were simulated in this paper. The simulation conditions of the two systems have no difference except temperature. The density changes of ·O_2_^−^ at different distances from the surface of phospholipid membrane in the two systems were obtained, as shown in Figure 2b and Figure 6.

It can be seen from Figure 2b and Figure 6 that the distance of the maximum density peak of ·O_2_^−^ in the system increased a little with the increase in temperature. It is at 1.8 Å and 2.0 Å for systems B (310 K) and C (330 K), respectively. According to statistics, the occurrence probability of ·O_2_^−^, $R_{•O_{2}^{-}}\;$(within 3.2 Å), decreased a little with temperature. The $R_{•O_{2}^{-}}$ values at 310 K (System B) and 330 K (system C) were 35.26% and 34.68%, respectively (Table 2). This phenomenon may be attributed that the enhancement of thermal motion counteracted part of the enrichment of ·O_2_^−^ near the membrane area.

### 2.6. Effects of Different Electrolyte Environments on ·O_2_^−^ Ions Density near the Lipid Membrane

**Figure 7:**
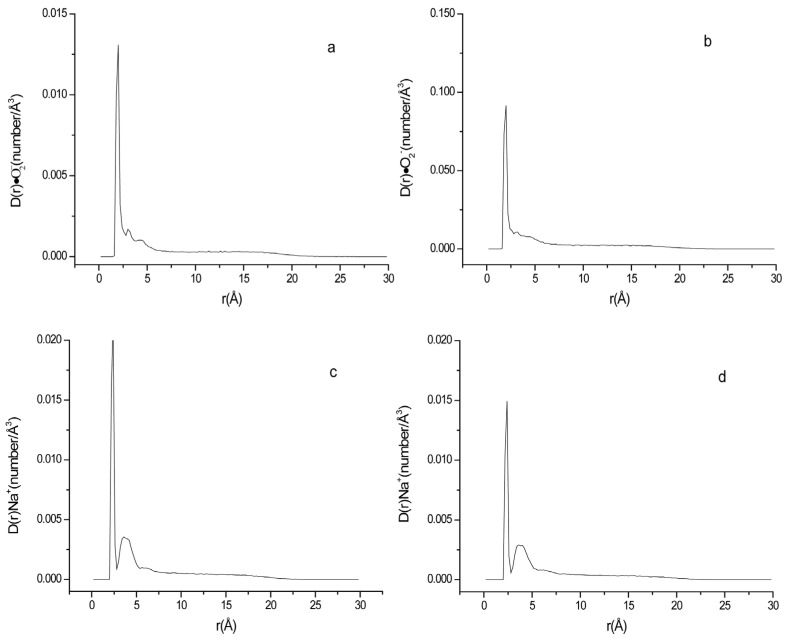
Density of ·O_2_^−^ and Na^+^ at different distances to the surface of phospholipid membrane. (**a**) system D ·O_2_^−^; (**b**) system E ·O_2_^−^; (**c**) system D Na^+^; (**d**) system E Na^+^.

The existence of electrolytes in the solution can influence the interaction between the target ions and membrane. Different electrolytes may have different effects. Some people have investigated the effect of different electrolytes on the lipid membrane structure and potential. Bockmann et al. [50] investigated NaCl on the lipid bilayer. They found that the self-diffusion of POPC lipids within the bilayer was decreased by the increase in NaCl concentration. Ganesan et al. [51] investigated the influence of monovalent cation (Na^+^, K^+^ and Li^+^) sizes on nanodomain formation in anionic-zwitterionic mixed lipid bilayers and found that phosphatidylserine (PS) lipid packing patterns depended on the cation size existed. Klasczyk et al. [52] studied the interactions of alkali metal chlorides with phosphatidylcholine vesicles and found that the order of the ion’s influence on the decrease in POPC membrane potential was Li^+^ > Na^+^ > K^+^ ≈ Rb^+^ ≈ Cs^+^. Since alkali metal cations with different sizes have different influence on the membrane potential, their influence on the anion distribution in the system may also be different. Thus, in order to examine the effect of different monovalent cations on the interaction of ·O_2_^−^ with membrane, the K^+^ in system A and B were replaced by Na^+^ to form systems D and E, and other simulation conditions were unchanged. The dynamic results were shown in Table 2 and Figure 7.

It can be seen from Figure 7 after replacing K^+^ with Na^+^, the value of $R_{•O_{2}^{-}}$ decreased from 56.89% to 51.61% (Table 2) in neutral membrane systems, while it increased from 35.26% to 42.31% in negatively charged lipid membrane systems. Compared with the neutral membrane system containing Na^+^ (System D), $R_{•O_{2}^{-}}$ was decreased by 18.02% ((51.61–42.31%)/51.61%) in the negatively charged system containing Na^+^ (system E). This relative difference of $R_{•O_{2}^{-}}$ between the neutral and negative charged membrane systems E and D was smaller than that of corresponding K^+^ systems B and A (38.02%). According to the investigation result of Klasczyk et al. [52], it can be speculated that the membrane ζ-potential in the presence of Na^+^ with small ion size is lower than that of K^+^ and the rejection to ·O_2_^−^ by the negatively charged membrane was less obvious in the presence of Na^+^. The negatively charged membrane has a lower repulsion to ·O_2_^−^s in Na^+^ system than that in K^+^ systems. On the other hand, the maximum density peaks for Na^+^ and K^+^ appeared at 2.4 Å and 2.8 Å from the membrane, respectively. It may be attributed to that the smaller radius of Na^+^ makes it have a strong interaction with the carbonyl oxygen groups of lipid [53] and allows it to squeeze closer to the membrane molecules. More cations near the surface of the membrane can more effectively counteract the repulsion of the negative charge of the membrane to the superoxide anion, which resulted in a higher density of ·O_2_^−^s near the negatively charged membrane than that of K^+^ as the equilibrium ion.

In animals and human bodies and tissues, there exists a high concentration of extracellular Na^+^ but an extra-high concentration of intracellular K^+^. Based on the investigation results above, a high K^+^ concentration environment in biological somatic cells is conducive to the protection of cell membranes and the maintenance of cell integrity. It was reported that the negative charge distribution of neutrophil membranes increases from the perinuclear area toward the plasma membrane [54], which may provide higher protection to the nucleus of neutrophil cells.

**Figure 8:**
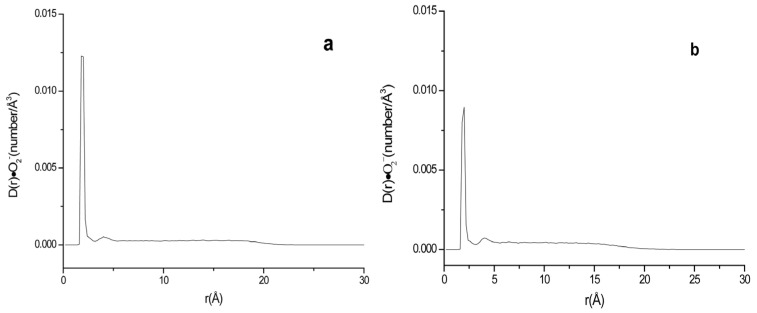
Density of ·O_2_^−^ at different distances to the surface of phospholipid membrane in systems F (**a**) and G (**b**).

In order to examine whether the concentration of the equilibrium electrolyte affects the distribution of superoxide anions, as a comparison, molecular dynamics equilibrium simulations were carried out for neutral and negatively charged phospholipid membrane systems with 0.15 M KCl, respectively. The new systems F and G were generated from systems A and B by adding extra 21 Cl^−^ and 21 K^+^ to make the concentration of KCl roughly 0.15 M. ·O_2_^−^ density distributions at different distances of the surface of lipid membrane in systems F and G are shown in Figure 8 and the R values are listed in Table 2.

For systems containing 0.15 M KCl, it can be seen that the $R_{•O_{2}^{-}}$ in system F was 50.79%, while that in system G was 39.10%. The $R_{•O_{2}^{-}}$ was reduced by 23.01% ((50.79–39.10%)/50.79%) in system G compared with that of the neutral membrane system F, which was lower than the 38.01% reduction in $R_{•O_{2}^{-}}$ of system B to system A. According to the research of Ohki [53], the addition of salt can lower the membrane potential. The existence of higher electrolyte concentration in negatively charged lipid membrane system G may partly counteract the membrane negative charge, therefore, it decreased the membrane repulsion to superoxide anions.

### 2.7. Effect of the Glycolipid on ·O_2_^−^ Ions Density near the Lipid Membrane

**Figure 9:**
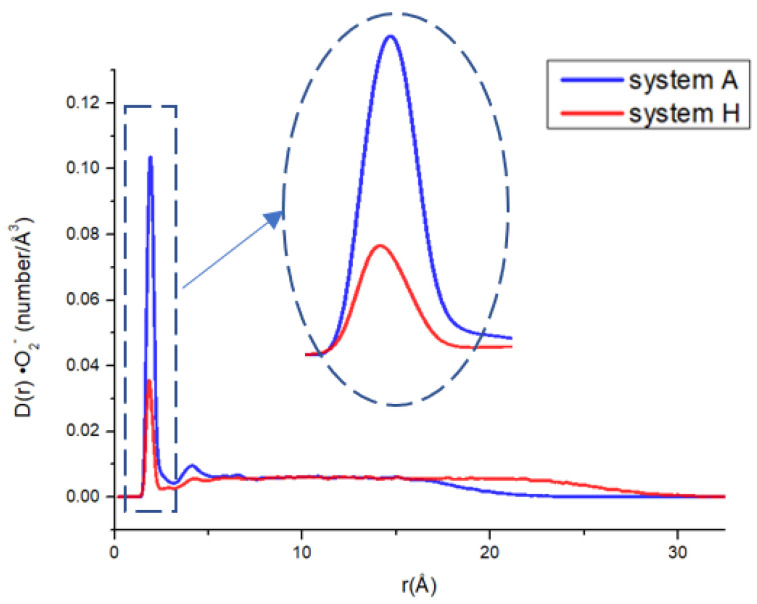
Density of ·O_2_^−^ at different distances to the surface of phospholipid membrane in system B and H (the glycosyl groups in glycolipid are excluded during the distance calculation).

To examine the effect of glycosyl on superoxide anion distribution near the lipid bilayer, the glycolipid bilayer, consisting of POPE:POPG:GM1:GD1a = 9:3:1:1 was constructed with Charmm-GUI with ·O_2_^−^ and K^+^ were added with Packmol. After molecular dynamic equilibrium, the D(r) of ·O_2_^−^ varies with the distance from the surface of the lipid bilayer (the glycosyl groups in glycolipid are excluded during the distance calculation because the distance of hydrocarbon chain to ·O_2_^−^ is important for lipid peroxidation) is shown in Figure 9 (for comparison, D(r) of ·O_2_^−^ changes with the distance from the surface of the lipid bilayer is also given).

It shows that the significant difference between system H and the previous systems without glycolipid is that $R_{•O_{2}^{-}}$ value of system H is much smaller (12.32%). It is only as much as 21.7% that of system A of the neutral membrane without glycolipids (it decreased by 78.3%). Due to the covering of glycosyl on the surface of the bilayer, ·O_2_^−^ could only be distributed in distant bulk solutions, therefore, it reduced the density of ·O_2_^−^ near the phospholipid bilayer, which may significantly reduce ·O_2_^−^ density near the lipid bilayer (excluding saccharide groups).

### 2.8. The Distribution of Different Components and Electrostatic Potential along the Vertical Direction of the Membrane Surface

**Figure 10:**
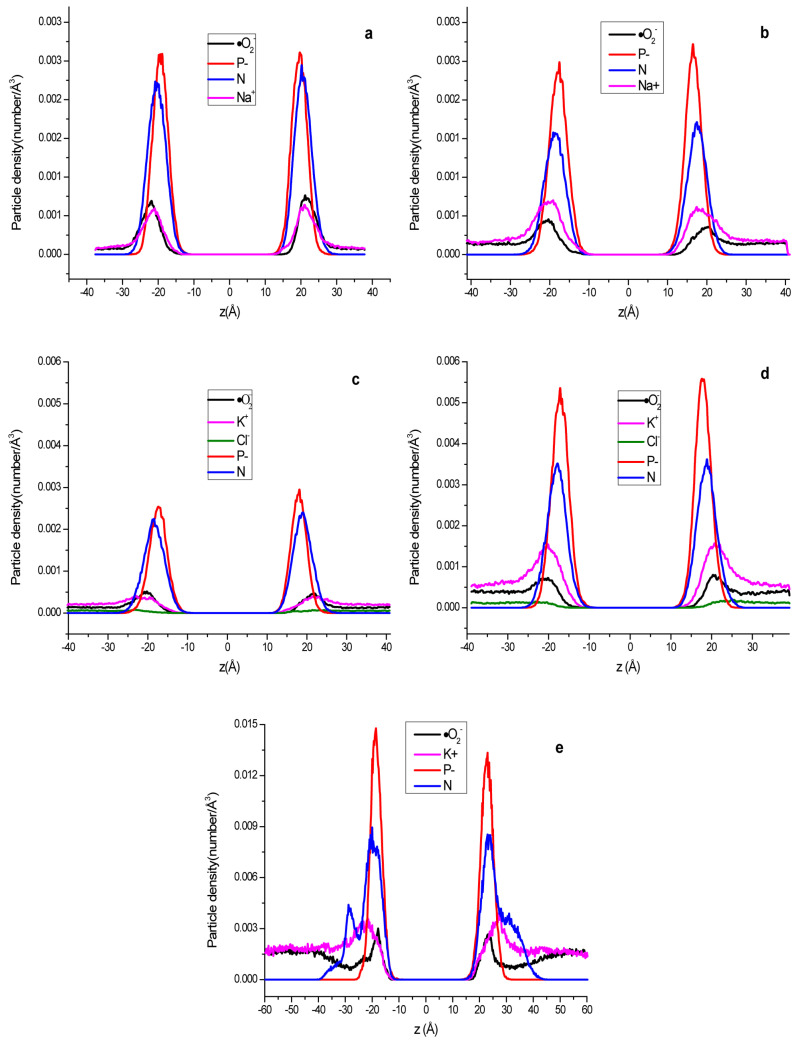
Density profiles across the lipid bilayer for systems D, E, F, G and H. Profiles for ·O_2_^−^, K^+^, Cl^−^, phosphate (P) and amine(N) in lipid. (**a**) neutral membrane system D; (**b**) negatively charged system E; (**c**) neutral membrane system F; (**d**) negatively charged system G; (**e**) negatively charged glycolipid system H.

In order to examine the distribution of different components along the direction perpendicular to the membrane plane (z-axis), some systems were selected to analyze the particle density distribution along the z-axis. It can be seen from Figure 10a–e the distribution of different components along the direction perpendicular to the membrane (z-axis) in system D, E, F, G and H, respectively. The distance between two phosphorus density peaks (Labeled P) can approximately represent the thickness of the membrane. The distances between the two P peaks are 39.4 Å, 35.6 Å,34.4 Å and 35.2 Å (Table 3) for the neutral (a: system D and b: system E) and negatively charged (c: system F and d: system G) systems, respectively. It can be seen that the thicknesses of neutral phospholipid bilayers (D and F) are larger than that of negatively charged phospholipid bilayers (E and G). This may be due to the fact that neutral POPE is actually amphoteric phospholipids, which have more polar atoms to make them more hydrophilic, reducing the interaction between the bilayers and thus thickening the membrane. There is no significant change in the distance between the peaks of ·O_2_^−^ and P, but the ratio of peak height to that of the bulk horizontal line Rph, which represents the ·O_2_^−^ appearance opportunity at the peak position and the bulk solution, is significantly reduced in the negatively charged membrane systems (Table 4), indicating that the occurrence of superoxide anions near the membrane is less than that of the neutral membrane systems. What is interesting is that not only the Rph of the glycolipid membrane system is very small (1.91), but it also appears two ·O_2_^−^ low-density regions near membrane (z = 31.6 Å and 93.2 Å) in the aqueous phase, which further reduces ·O_2_^−^ density near the phospholipid bilayer. The distribution of N atoms which represents that of the protonated amine groups in phospholipid molecules of D, E, F and G was also calculated. The two peaks of N density appeared to the water phase side compared with that of P with wider distances of 40.0 Å, 37.4 Å, 37.4 Å and 36.6 Å (Table 3) for systems D, E, F and G, respectively, indicating the protonated amine groups tends to stay in water more than phosphate anions, which is consistent with the research results of Pandit and Berkowitz [55]. For system H, because the glycolipids have extra acetylated amines on their glycosyl groups, the N density curve has two extra peaks in the aqueous phase. It can be seen that both anions such as ·O_2_^−^ and cations (K^+^ and Na^+^) have a maximum density peak near each membrane surface, respectively, representing their density in these areas higher than the bulk concentration, which is consistent with the phenomenon described in the literature [56]. In the phospholipid bilayer membrane systems D, E, F, G and H, the distances between corresponding density peaks of different components such as atoms P, N, ·O_2_^−^, K^+^(or Na^+^), Cl^−^ on both sides of the bilayer are shown in Table 3.

**Figure 11:**
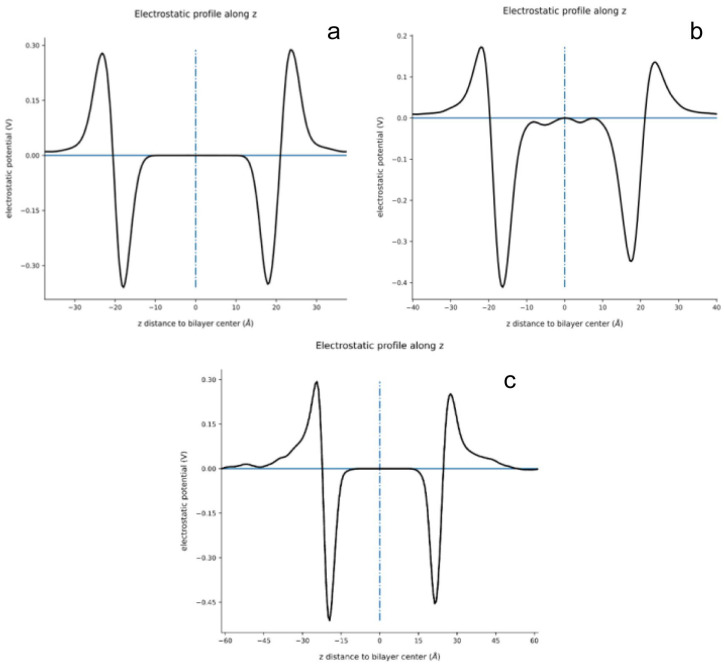
Electric potential along the Z-axis of systems A (**a**), B (**b**), and H (**c**).

The membrane potential has an important influence on the ion distribution near the membrane. Therefore, the potential distribution of neutral and negatively charged membrane systems was calculated by cgpotential_vol.py python program (written by Jean Helie, https://github.com/jhelie/cg_potential_vol, accessed on 1 March 2023) based on MD analysis library [57]. Figure 11 and Table 5 shows the change of electric potential along the Z-axis of systems A, B and H. It can be seen that there exist a positive potential area near both the neutral and negatively charged membranes even for the glycolipid membrane in the aqueous phase side due to the protonated amines, which is consistent with the research of Pandit et al. [55]. However, the significant low positive potential near the negatively charged membrane surface may decrease the attraction of the membrane to ·O_2_^−^ with the opposite charge, which is one of the reasons for the comparatively low ·O_2_^−^ density near the negatively charged membrane.

This study focused on investigating the distribution of superoxide anions and other electrolyte ions near typical neutral and negatively charged phospholipid bilayer systems using molecular dynamics simulations. The aim was to explore how membrane charge affects the diminished lipid peroxidation ability caused by the repulsion of superoxide anions. However, it is important to note that real biological systems are far more complex than the model systems used in this study. Further experimental validation is required to understand the actual events occurring near phospholipid bilayers in biological systems.

## 3. Materials and Methods

### 3.1. Model Preparation

In order to examine the effect of some factors such as membrane charge, electrolytes, temperature and sugar components on the density of ·O_2_^−^ near phospholipid bilayer, palmitoyl-oleoyl-phosphatidyl-ethanolamine (POPE) was used to construct the neutral phospholipid bilayer (in fact, most neutral phospholipids are amphoteric phospholipids because they contain equal number cationic group such as –NH^3+^ and anionic group such as phosphate diester group). The number ratio of neutral phospholipid POPE and negatively charged phospholipid palmitoyl-oleoyl-phosphatidyl-glycerol (POPG) (ne:ng = 3:1) was used to construct negatively charged bilayer. The gangliosides GM1 and GD1a (molar ratio 1:1) were used to construct the glycolipid membrane (POPE:POPG:GM1:GD1a = 9:3:1:1). CHARMM-GUI webserver [58] was used to produce the phospholipid bilayer systems (some have gangliosides) with membrane size of 80 Å × 80 Å. The bilayer systems were then converted to AMBER format using the Python program charmmlipid2amber.py in AmberTools14 [59]. The rectangular boxes having the sizes of 80 Å × 80 Å × 90 Å (non-glycolipid systems) and 80 Å × 80 Å × 120 Å (glycolipid containing system) containing the lipid bilayer, water, counter-ions and electrolyte was constructed by using Packmol [60] with the phospholipid bilayer constructed above located at the xy plane. The superoxide anion, whose structure was pre-optimized by Gaussian09 at the B3LYP/6–31 + g (d, p) level [61], was added to the box by the Packmol program. The counter ions such as K+ or Na+ were added according to the number of phospholipid POPG (with negative charge) and superoxide anions so that the system is electrically neutral. The remaining space is filled with solvent water using the tleap program in AmberTools16. Extra KCl or NaCl electrolytes were added in some systems at a proportion of about 150 nM to balance the total charge in the systems and to mimic the general ion concentration in mammalian cells. For the parameterization of the systems, the Amber Lipid17 force field [62] combined with the Amber ff14SB force field [59,63], TIP3P parameters for water [64] and the Li/Merz ion parameters [65] were employed. For the superoxide anion, its force field parameters can be obtained by using AmberTools combined with Gaussian 09 quantum chemistry calculation, just as many literatures described [66,67]. The Amber Gaff force field [68] was used to parameter superoxide anion and the RESP charges obtained from quantum chemistry computation of Gaussian09 at B3LYP/6–31 + G (d, p) level with pop = mk iop (6/33 = 2) iop (6/42 = 6) iop (6/50 = 1) were applied to atoms of superoxide anions. The inpcrd and prmtop files used for Amber molecular dynamics were finally constructed using tleap program. Finally, the input files for the MD in Gromacs 5.02 (top and gro files) were obtained from the conversion of the inpcrd and prmtop of Amber MD input files using Acpype.py program [69].

### 3.2. MD Simulation

There are 8 systems (the composition shown in Table 1) that were used for MD research. All MD simulations were carried out using Gromacs 5.02 [56] (http://www.gromacs.org/, accessed on 1 March 2023) [70]. For each system, energy minimization was executed using the steepest-descent method for 150,000 steps and a 5 ns MD simulation using an NVT ensemble [70] was then performed by gradually heating the system to the temperature needed. Subsequent MD simulation with an isothermal-isobaric (NPT) ensemble [70] to equilibrate the density at 1 atm was then carried out. The time step for NVT and NPT ensembles are all 1fs. In all equilibrium simulations, short-range electrostatic interactions are applied and Van der Waals force had a 1.4 nm cutoff. The long-range interactions were calculated using the Particle Mesh Ewald (PME) algorithm [71] and the leapfrog MD integrator with steps of 0.001 ps. The total equilibrium time for 8 systems was listed in Table 1. Finally, the production stage was run for 5 ns for every system. During the equilibrium and production run, the temperature was kept constant at a preset temperature using the Nose–Hoover thermostat with time constant 0.1 ps, while pressure was maintained constant using the isotropic Parrinello–Rahman barostat [72] with a time constant of 1 ps. A LINCS algorithm [73] was used for the constrain of all bonds in the system to make it possible to run a longer time step.

### 3.3. Data Processing

The movement of the phospholipids and gangliosides of the bilayer during the simulations ware analyzed through the calculation of the root mean square deviation (*RMSD*) of the lipids using RMSD Visualizer Tool (V 1.0) in VMD1.91 extensions. This tool is a VMD extension available at https://www.ks.uiuc.edu/Research/vmd/plugins/rmsdvt/ (retrieved on 8 January 2021). The system is considered as a stable or a metastable state when RMSD oscillates around a constant value. The RMSD of the lipids from their initial positions in the system by least-square fitting the structure to the reference structure using the equation [74]:

(8) $$RMSD=\sqrt{\frac{\sum_{\alpha =1}^{N\alpha }{\left({\vec{r}}_{\alpha }\left(t_{j}\right)-\langle {\vec{r}}_{\alpha }\rangle \right)}^{2}}{N_{\alpha }}}$$

where *Nα* is the number of atoms whose positions are being compared, *Nt* is the number of time steps over which atomic positions are being compared, ${\vec{r}}_{\alpha }\left(t_{j}\right)$ is the position of atom *α* at time *tj*, and (${\vec{r}}_{\alpha }$) is the average position value of atom *α* to which the positions ${\vec{r}}_{\alpha }$(*tj*) are being compared. Finally, it is defined as:

(9) $$\langle {\vec{r}}_{\alpha }\rangle =\frac{1}{N_{t}}\overset{N_{t}}{\underset{j=1}{\sum }}{\vec{r}}_{\alpha }\left(t_{j}\right)$$

Thereafter, 5 ns of the dynamics trajectory with 1000 frames were used for the statistics of occurrence of superoxide anion near the lipid membrane surface. A Linux shell program written by us was used to sum the superoxide anion numbers near the lipid membrane surface.

The figures about the density changes of the components with axis in membrane systems such as ·O_2_^−^, K^+^, Na^+^ and Cl^−^ were sketched by Origin 7.5 and the images about the arrangement of the components of the lipid membrane systems were made by Biovia Discovery 2016 Client [75,76]. The electrostatic potential profiles along Z-axis were exported by cgpotential_vol.py python program (written by Jean Helie, https://github.com/jhelie/cg_potential_vol, accessed on 1 March 2023) based on MD analysis library.

In order to compare the density of ·O_2_^−^ and other components such as K^+^, Na^+^ and Cl^−^ at different distances from the surface of the phospholipid bilayer, their density distribution at different distances, *D*(*r*), from the surface of the phospholipid bilayer within the last 5 ns time step after the systems were equilibrated. *D*(*r*) was calculated as the following equation:

(10) $$D\left(r\right)=\frac{N\left(r\right)}{D_{x}\times D_{y}\times \delta \left(r\right)\times f}$$

where r is the distance between the particles (·O_2_^−^, K^+^, Na^+^ and Cl^−^) and the phospholipid molecule of the bilayer. *N*(r) is the number of stated ions in the slices between r and r + δr along the lipid surface, which is collected from the 5 ns of the dynamics trajectory with 1000 frames. *f* is the frames for the dynamics trajectory for data collecting. *D*x and *D*y are the lengths of the water box for molecular dynamics simulation along the direction of the phospholipid bilayer membrane plane. The data were collected and processed using a tcl language program developed by us and running in VMD software. The working principle of the program involves calculating the number of target particles within a distance range from r to r + δr from each frame in the trajectory file. Then, the target particle counts from all frames in the trajectory file are summed to obtain *N*(r). The range of r is from 0 to 40 Å. In this study, δr is set to 0.2 Å.

## 4. Conclusions

As an anion radical, ·O_2_^−^ is widely distributed in the body and can be converted into other ROS species involved in lipid peroxidation. Investigating the factors that influence its density distribution near the phospholipid membrane is of significant importance in preventing or mitigating lipid peroxidation. To examine the impact of membrane charge, temperature, aqueous electrolytes and the presence of glycolipids on the distribution of ·O_2_^−^ near the phospholipid membrane, this study constructed eight different neutral and negatively charged phospholipid membrane systems containing superoxide anions.

By comparing the $R_{•O_{2}^{-}}$ values near negatively charged and neutral phospholipid membranes, it was observed that the probability density of ·O_2_^−^ near the negatively charged membrane surface was significantly reduced. This can be attributed to the electrostatic repulsion between the negative charge of the membrane and the negatively charged ·O_2_^−^, which weakens the density distribution of ·O_2_^−^ near the negatively charged phospholipid membrane. Considering that ·O_2_^−^ can convert to hydroxyl radicals, which are capable of inducing lipid peroxidation, this reduction in density distribution subsequently decreases the probability of lipid peroxidation occurrence.

From the results of molecular dynamics simulations at different temperatures, specifically 310 K and 330 K, it was found that at higher temperatures, the difference between ·O_2_^−^ near the negatively charged and neutral membranes decreased. This phenomenon may be attributed to the enhanced thermal motion, which partially offsets the influence of membrane charge on the density distribution of ·O_2_^−^ near the membrane surface.

Additionally, the influence of different electrolytes, namely NaCl and KCl, on the distribution of ·O_2_^−^ near the phospholipid membrane was investigated. It was observed that compared to Na^+^, the reduction in ·O_2_^−^ near the negatively charged phospholipid surface was more significant in the presence of K^+^ as the counterion under equilibrium conditions, indicating high K^+^ concentration environment in biological somatic cells is conducive to the protection of cell membranes and the maintenance of cell integrity.

Furthermore, by molecular dynamics simulations on phospholipid membranes containing glycolipids, it was observed that the presence of sugar moieties on the membrane surface significantly reduced the density distribution of ·O_2_^−^ near the membrane. This finding highlights the important role of glycolipids in protecting phospholipid molecules and reducing lipid peroxidation.

Finally, the membrane potential calculation showed that the significant low positive potential near the negatively charged membrane surface may decrease the attraction of the membrane to ·O_2_^−^ with opposite charge, which may be one of the reasons for the comparatively low ·O_2_^−^ density near the negatively charged membrane.

However, it should be noted that this study only examined the impact of membrane charge and glycosyl group coverage on the distribution of superoxide anion radicals and their potential protection against lipid peroxidation using theoretical MD method. These findings need further experimental research for validation, such as determining the concentration differences of ·O_2_^−^ near membranes with different charges. In actual systems, lipid peroxidation is influenced by multiple free radicals and antioxidative mechanisms, which require comprehensive consideration from various perspectives. The approach used in this study can be applied to examine the effects of negatively charged phospholipid membranes on the density distribution of other charged free radicals, such as •ONOO^−^ or charged antioxidants such as glutathione near the lipid surface.

## Figures and Tables

**Table 1:** The composition and temperature for the 8 systems investigated.

System	POPE	POPG	·O_2_^−^	K^+^	Na^+^	Cl^−^	T(K)	GM1	GD1a
A	√	-	80	80	-	-	310	-	-
B	√	√	80	134	-	-	310	-	-
C	√	√	80	134	-	-	330	-	-
D	√	-	80	-	80	-	310	-	-
E	√	√	80	-	134	-	310	-	-
F	√	-	80	101	-	21	310	-	-
G	√	√	80	155	-	21	310	-	-
H	√	√	80	140	-	-	310	√	√

“√”: Represents that the system contains this component; “-”: Represents none.

**Table 2:** The probability of the ions near the phospholipid membrane surface (Area of peak 1 to the total area of ions in the systems).

System	R (%)			
	·O_2_^−^	K^+^	Na^+^	Cl^−^
A	56.89	21.97	-	-
B	35.26	23.83	-	-
C	34.68	29.45	-	-
D	51.61	-	41.18	-
E	42.31	-	32.95	-
F	50.79	16.46	-	18.16
G	39.09	25.16	-	21.39
H	12.32	20.14	-	-

**Table 3:** The distance between the two density peaks of different species in the systems D–H (Å).

System	P	N	·O_2_^−^	Na^+^/K^+^	Cl^−^
D	39.4	40.0	43.2	42.0	-
E	34.4	37.4	40.6	39.0	-
F	35.6	37.4	42	44.2	44.8
G	35.2	36.6	41	42	48.8
H	41.4	43.0	41.4	48.6	-

**Table 4:** Rph of the systems (D–H).

System	D	E	F	G	H
*R*_ph_	9.87	3.65	2.92	2.0	1.91

**Table 5:** Data related to the membrane potential of systems A, B and H.

System	Maximum Positive Potential (×10^−2^ V)	Maximum Negative Potential (×10^−2^ V)	Distance between the Positive Potential Peaks (Å)	Distance between the Negative Potential Peaks (Å)
A	28.7	−35.9	46.68	35.76
B	17.2	−41.1	45.92	33.85
H	9.5	−51.1	51.85	41.35

## Data Availability

The data used to support the findings of this study are available from the corresponding author upon request.
